# Tomato-Processing By-Product Combustion: Thermal and Kinetic Analyses

**DOI:** 10.3390/ma12040553

**Published:** 2019-02-13

**Authors:** Besma Khiari, Marwa Moussaoui, Mejdi Jeguirim

**Affiliations:** 1 Industrial Engineering Department, National School of Engineering of Carthage, 45, Avenue des Entrepreneurs, Charguia 2, Tunis 1002, Tunisia; besmakhiari@yahoo.com (B.K.); marouamoussaoui@yahoo.com (M.M.); 2 Institut de Science des Matériaux de Mulhouse (IS2M), Université de Strasbourg, Université de Haute Alsace, UMR CNRS 7361, Mulhouse CEDEX 68093, France

**Keywords:** tomato waste, kinetics, thermal analysis, combustion

## Abstract

This paper is part of a sustainable development approach, the aim being to develop a thermochemical energy recovery path while reducing the amount of tomato waste issued from agro-industrial units. The thermal process may contribute to an environmentally friendly management and help tomato processing industries creating new economic profitable circuits in an increasingly competitive context. The adopted approach was to follow the operating conditions needed for a complete thermal degradation through a thermal and kinetic analyses. The results of the tomato waste characterization confirmed their suitability to a thermochemical processing with high volatiles and fixed carbon and interesting high heating values comparable to sawdust biomass. We were able to isolate of the decomposition domains and extract kinetic parameters. Three kinetic models were applied; Flynn–Wall–Ozawa (FWO) simulated the best the combustion process. Calculated curves were validated by the first order (n = 1) model except for the slow heating rate of 5 °C/min which was fitted by the contracted cylinder model. The conclusions of this paper could help in optimizing the combustion process in order to achieve high energy recovery from tomato residues. Obtained kinetic data would help in the design of combustion reactors.

## 1. Introduction

Tomato (*Solanum lycopersicum* L.) is a fleshy fruit, originally from northwestern South America, but is widely consumed in many countries, fresh or processed. According to statistics from the Food and Agriculture Organization of the United Nations, world tomato production is steadily increasing from 64 Mtons in 1988 to more than 100 Mtons, with China being the world’s largest producer followed by the United States, Turkey, India, Egypt, Italy and Iran. More than 30% of these quantities are processed (dried, concentrated, frozen, etc.). This leads to the generation of a variety of by-products and wastes including pulp, skins and seeds, which account for 3% of the fresh tomato i.e., about 0.9 million tons. Moreover, as tomatoes are processed for long periods, the daily production of the residual rate is very high. Tunisia is ranked the eleventh producer, but it is the first world consumer of concentrated tomatoes. In fact, tomato cultivation in Tunisia covers an average area of 29,000 ha/year, with an average production of around 1.2 million tons. This production comes from field crops, i.e., seasonal and late tomatoes (mainly in the west, north west and north east) and crops grown under shelter, i.e., cold greenhouse, also called glasshouse (in the center of the country) and greenhouse heated by geothermal waters (exclusively in the eastern south). The varieties include Rio Grande, Durinta, Tylka, Abigaile, etc. 

Tomato waste constitutes 90% of fruit and vegetable canning waste with more than 35 thousand tons per year [1]. Seasonal discharges and centralized tomato waste from agri-food industries have negative effects on the environment due to high humidity (over 60%), low pH, high contents of phosphorus, potassium, organic and phenolic phytotoxic and antibacterial substances, which makes them resistant to biological degradation [2]. Despite the low cost, tomato waste-based fertilizers are declared not cost-effective [3]. Further available technologies of tomato waste valorization include pharmaceutical and cosmetic usage but the corresponding by-products meet the same first problematic. It is, therefore, essential to find alternative practices for better management [4]. According to a thorough literature review, management by thermochemical valorization was rarely addressed. In fact, the energy conversion of tomato waste thermochemically combines several processes based on the cracking of biomolecules under the effect of heat [5].

Combustion is the process currently exploited at industrial scales. Nevertheless, the economic and environmental issues related to the exploitation of these processes make industrial facilities vulnerable [1]. Several parameters must be taken into account during the plant design, namely the fuel characteristics (water content, particle size, density, temperature, etc.), fuel injection and its frequency, minimum storage autonomy, etc.

Table 1 summarizes some physical and chemical properties of tomato waste met in literature. The high volatiles and the high levels of carbon, hydrogen and oxygen suggest the high potential for energy recovery of these residues by pyrolysis, gasification or combustion.

The elemental composition also made it possible to calculate the (H/C) and (O/C) ratios of a given hydrocarbon. These ratios ranged from 12.9 to 14.9% and from 37 to 78.6%, respectively, which confirms again the applicability of a thermal degradation process. However, low mineral content and mass densities limit direct use as a biofuel. This is why some researchers have opted for the preparation of pellets or pellets in order to densify these residues in order to optimize energy production. In fact, the contents of mineral salts such as nitrogen, potassium, chlorine, calcium and heavy metals such as iron and manganese are in small quantities in the various works. For sulfur, the quantities obtained are small in comparison with those found in conventional fossil fuels. This result is interesting from an environmental and technical point of view.

Just like most of the lignocellulosic materials [10], tomato residues are made of three major constituents, namely cellulose (28.8%), hemicelluloses (19.4%) and lignin (8.2%). Hemicelluloses content is close to that of grape marcs [11] whereas cellulose percentage is not far from that of olive cakes [12]. Contents of fatty acids of those three agro-food industrial by-products, obtained by gas chromatographic analysis, are reported in Table 2 for comparison.

Fatty acids are common in vegetable oils (grapes, corn, sunflower, etc.) and are predominant in tomato waste. The oil content is estimated to be between 19.5 and 25.7 %W/W db by Mangut et al. while Rossini et al., found that it was around 21% [13]. Unsaturated fat in olive seeds is mainly composed of monounsaturated fatty acids (C16 and C18 make up over 96% total fatty acids), while polyunsaturated fats are the major acids in grape seed oil. The oil extraction processes carried out on several samples of tomato seeds showed that the latter have a mass fraction of unsaturated fatty acids greater than 80%, in particular linoleic acid (C18).

Gonzalez et al. have pelletized tomato waste to optimize the fuel feed of a 11.6 kW mural boiler for domestic heating. For 92.4% boiler efficiency, the optimum mixture was 75% of tomato residues and 25% of forest wastes [16]. Font et al. carried out experiments of tomato waste at three heating rates (5, 10, 20 K·min^−1^). The initial small endothermic peak was attributed to the evaporation of the humidity while the subsequent three small exothermic peaks were due to oxidative pyrolysis. The significant exothermic process and the two final small fractions observed in the last part of the decomposition were due to the combustion of char or carbonaceous residue [17].

## 2. Materials and Methods

Tomato waste used in this study was collected from a tomato-processing company named “Jouda”, located in Kairouan (Center of Tunisia). The samples were fresh with 60% db water content but were dried until moisture was 10%. Biomass solid fuels have different physicochemical characteristics, the determination of which is an important step prior to any energy recovery pathway.

### 2.1. Thermochemical Characterization

Physical and chemical analyses as well as High Heating Value (HHV) and Low Heating Value (LHV) were carried out according to the standards in Table 3. Elemental analysis was carried out with CHONS analyzer (ThermoFisher Scientific, Villebon, France).

### 2.2. Thermogravimetric Analysis

Heating effects on tomato waste result in mass loss and heat exchange. The evolution of mass loss can be followed by thermal analysis. In the present work, tests were conducted with Thermal Gravimetric Analysis coupled with a Differential Scanning Calorimeter (TGA/DSC3) + (Mettler-Toledo, Columbus, OH, USA) at different heating rates (5, 10, 20 and 30 °C/min) from room temperature to 950 °C. Each test was repeated at least three times.

### 2.3. Kinetic Models

The progress of the thermal degradation reaction is accompanied by various physico-chemical events such as crystallographic structure destruction, chemical bonds breaking, solid product recrystallization, gases desorption, etc. [18]. The reaction rate equation is then formalized by considering the geometry and the global kinetics of progression of the reaction interface [19,20]. The degradation of tomato waste is simplified by the following reaction mechanism:

$$\text{tomato waste}\;\overset{k}{\to }\;\text{X solid residue}+(1-X)\;\text{volatiles}$$

The fundamental rate equation usually applied is:

(1) $$\frac{\mathrm{dX}}{\mathrm{dt}}=k\left(T\right)f\left(X\right)$$

where k is the rate constant and f(X) is the reaction model that describes the reaction mechanism. The conversion rate X is given by Equation (2):

(2) $$X=\frac{W_{0}-W_{t}}{W_{0}-W_{f}}$$

where W_t_, W_0_, and W_f_ are time t, initial and final weights of the sample, respectively.

The rate constant k is given by:

(3) $$k=\mathrm{Aexp}\left(\frac{-\mathrm{Ea}}{\mathrm{RT}}\right)$$

where Ea is the activation energy (kJ/mol), R is the universal gas constant (8.314 J/K mol), A is the pre-exponential factor (s^−1^) and T is the absolute temperature (K).

If the heating rate, designed by β is constant and by applying Ln, one can write:

(4) $$\mathrm{Ln}\frac{\mathrm{dX}}{f\left(X\right)}=\frac{A}{\beta }\exp\left(\frac{-\mathrm{Ea}}{\mathrm{RT}}\right)\mathrm{dT}$$

Several kinetic models have been proposed, according to the mechanism described by the decomposition reaction (Table 4).

Kinetics can be determined in isothermal conditions (plot of X vs. t or dX/dt vs. t at constant temperature) or in non-isothermal conditions (plot X vs. T or dX/dt vs. T while gradually increasing the temperature). Three variants of this last method are usually used: Friedman, Flynn–Wall–Ozawa (FWO) and Kissinger–Akahira–Sunose (KAS) (Table 5).

## 3. Results and Discussions

### 3.1. Characterization Results

Proximate analyses and energy contents as well as the ultimate analysis are given in Table 6.

Results show that tomato waste may be an interesting source of energy, even though the power content is a little smaller than that of fossil solid fuels. The MV/CF ratio (9.5) being more than 4, tomato residues might be considered as very reactive [24].

### 3.2. Thermal Analysis Results

**Figure 1:**
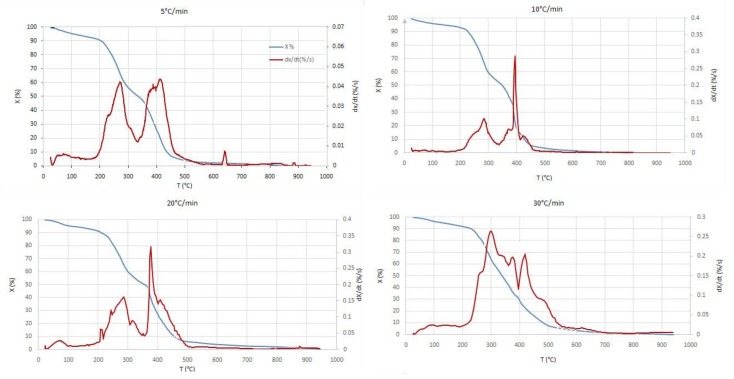
Profiles of tomato waste combustion for different heating rates.

The results of the thermal analysis of the thermal degradation of tomato waste in air, allowed us to follow the evolution at different heating rates (Figure 1). The thermal degradation of tomato waste is composed of three stages:

- a small loss weight stage corresponding mainly to drying;
- a large weight loss phase in which volatiles matter departure is assumed;
- a final step of mass loss which tends to be constant (char oxidation and ash formation).

More precisely, in the beginning of the combustion process, the particle heats up and the moisture in the sample is removed [25]. With the rise in temperature, pyrolysis takes place. A cloud of volatile matter is formed around the particle. This cloud may contain oxidizable materials [26]. The duration of this stage is generally very short (volatile matter contains a significant fraction of the heating value of the fuel). Once the volatiles are exhausted, the oxygen of the oxidizing gas (air) can then reach the surface of the solid residue [27]. The heterogeneous combustion phase then begins and lasts for a long time during the combustion process.

The evolutions of the mass loss profiles are very similar and close to each other, regardless the heating rate. We observe a first mass loss of about 5 to 10% (related to the evaporation of the moisture) between room temperature and 100 °C, before the mass stabilizes. From 200 °C to 430 °C, we notice a large loss of mass, up to 85%. Afterward and until 950 °C, the mass loss is very slow and seems to stabilize. Ash residue of 5% of the initial mass is obtained at the end of all tests.

From the different plots, one can state that increasing the heating rate results in higher temperature, broader combustion ranges and increasing maximum mass loss rate. One can also see that the complete combustion starts around 550 °C in the different curves. Beyond this temperature and up to 950 °C, no additional weight loss is recorded. However, a supplementary mass loss of about 1% is recorded, notably for 5 °C/min. This loss is attributed to ash evaporation combined with calcination. Indeed, the sample holder crucible is almost empty at the end of the experiments.

In general, the devolatilization temperature range and amplitude depend on the starting organic compound. The obtained results show that the combustion of Tunisian tomato waste occurs at temperatures lower than those found in literature. This behavior is very clear especially during the devolatilization phase and is explained by the difference in texture and climatic conditions of plantation. In addition, the temperature ranges and the intensity in which the combustion takes place depend on the nature of the waste to be degraded. For example, the degradation of volatiles occurs at low temperatures (135–325 °C) for sawdust, while that of present Tunisian tomato waste at high temperatures (225–650 °C) and from 470 to 800 °C in the work of Font et al. [17].

### 3.3. Kinetic Results

**Figure 2:**
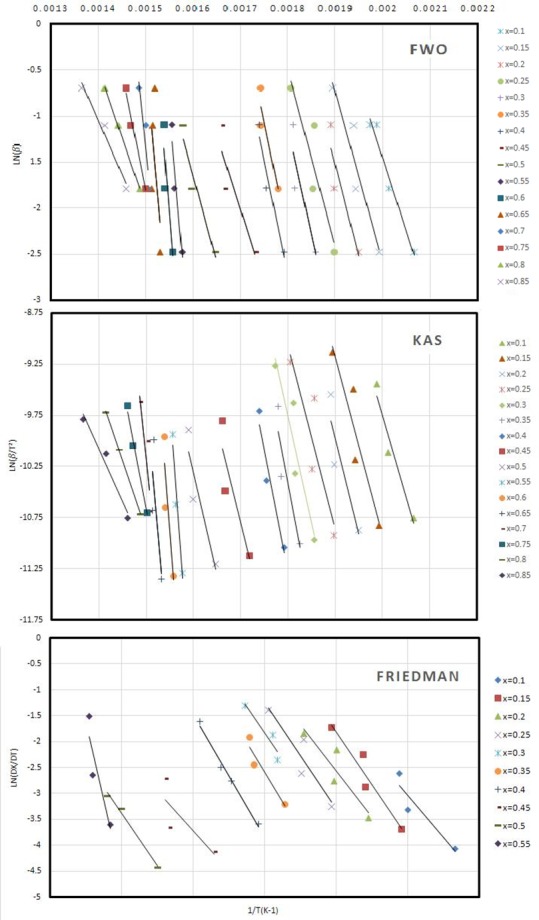
FWO, KAS and Friedman kinetic linear diagrams.

Isoconversion methods were applied at four different heating rates (5, 10, 20 and 30 °C/min). Linear diagrams of KAS, FWO and Friedman are shown in Figure 2. Since the initial loss of mass is attributed to moisture loss, the first conversion rate selected to determine the kinetic parameters is 10% while the last point is taken at 85% for KAS and FWO and at 55% for Friedman. Above this value, no good correlation coefficient is obtained.

**Figure 3:**
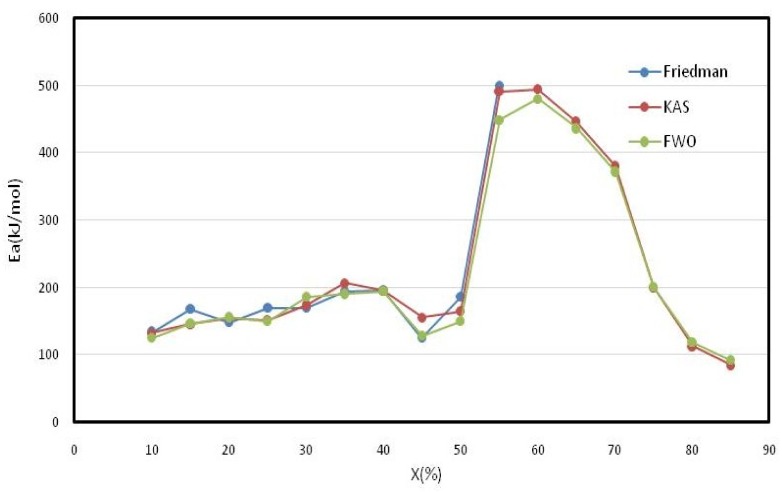
Activation energy variation according to KAS, FWO and Friedman models.

The corresponding calculated values are presented for each conversion rate in Table 7. The average activation energy values for tomato waste using the KAS, FWO and Friedman methods are, respectively, 231.21, 223.86 and 199.31 kJ/mol. The activation energy increases as the conversion rate increases until conversion rates of around 55% and 60% are achieved, recording maximum values of 494.96, 480,893 and 500.47 kJ/mol. Beyond these rates, there is a drop in activation energy values as the conversion rate increases until the end of the degradation process (Figure 3).

KAS and Friedman are very close while FWO is slightly off. The activation energy Ea calculated at different conversion rates can be attributed to the decomposition of macro-components contained in the tomato waste. At low conversion levels (10–30%), activation energies between 169.62 and 186.46 kJ/mol are probably related to hemicelluloses degradation. Activation energies continue to increase to 190.40–206.92 kJ/mol. These energies can be attributed to the cracking of cellulose and are close to values met in literature around 150–250 kJ/mol [28,29]. Since lignin is the most stable compound, its degradation occurs over wide intervals [8]. In our case, lignin degradation occurs between 55% and 75% conversion rates with maximum Ea. Finally, beyond these rates, i.e., during char combustion, energy values tend to decrease until the end of the combustion process.

**Figure 4:**
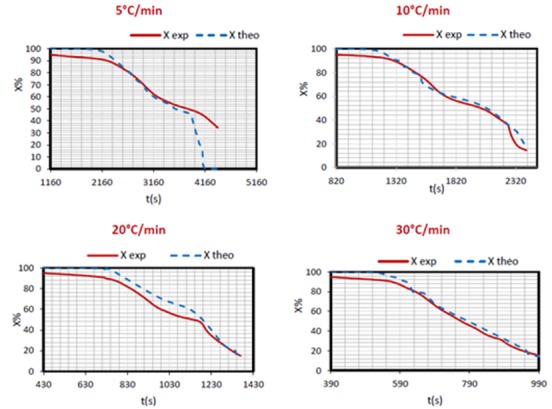
Mass loss fraction validation profiles.

To validate these results, the present study suggests the evaluation of the evolution of the conversion rate and theoretical masses compared to experimental ones (Figure 4).

As mentioned earlier, the choice of f(X) depends on the reaction order and on the model used. In most biomass thermal degradation works, the chosen kinetic model is first order (n = 1), even if each phase could be described a different model.

Since FWO method provided the best correlation coefficients, the corresponding results were adopted while using the first-order kinetic model for different heating rates (5, 10, 20 and 30 °C/min), Experimental and theoretical curves have almost the same profiles. A slight difference (4%) is noticed during the drying phase. This difference tends to cancel out in the active pyrolysis and oxidation phases at 10 and 30 °C/min but is still visible in the active pyrolysis and vanishes during the oxidation stage at 20 °C/min. This could be due to errors related to the uncertainties of the chosen models or to experimental errors. As for 5 °C/min heating rate, the curves obtained experimentally and numerically show a slight difference, not more than 4% during the drying step, but good agreement is recorded during the active pyrolysis phase. Beyond this phase, conversion rates are underestimated by the first-order model.

**Figure 5:**
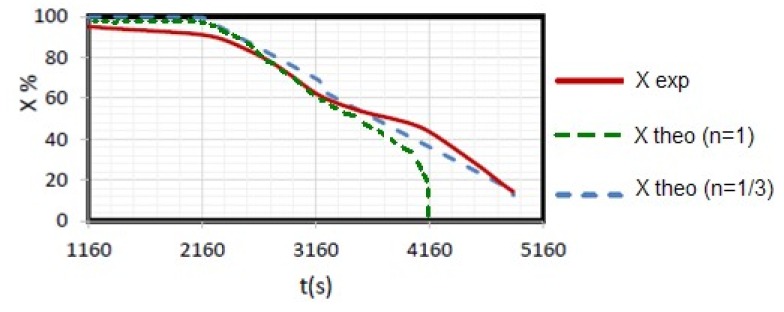
Model validation for 5 °C/min heating rate with n = 1/3 and n = 1 kinetic models.

As the first-order kinetic model did not describe the combustion process for the slow pyrolysis (5 °C/min), another simulation with n = 1/3 was carried out. The corresponding plot with f(X) = (1−X)^1/3^ is seen in Figure 5. The fitting is better, despite some deviations.

## 4. Conclusions

The great quantities of generated tomato waste as well as the scarcity of works dealing with their thermochemical degradation were motives to carry out this work. The characterization of the tomato residues showed their suitability for combustion processes. Our results showed that the oxidation operation is a complex reaction involving several mechanisms. In order to offer a decision support to develop a recovery process in biofuel, biogas and bio char while meeting both environmental and economic requirements, combustion was run at different heating rates. Kinetic parameters could then be extracted and the kinetic model constructed. The fittings were good and the models validated by first-order or contracted sphere models according to the applied heating rate.

Based upon the total quantity and the HHV of the tomato waste, and assuming a total recovery, the energy recovered is around 16.3 kTOE (tons oil equivalent); that is around 6‰ of the deficit in the energy balance of Tunisia. However, this calculation is rough and very approximate. Indeed, the main failing is that the input is presented in the form of a lot of small quantities spread out over a large area (30 factories), making the aggregation challenging or uneconomic, even if these units are all located in the Cap-Bon region (North East) in Tunisia. In this case, the tomato waste may need to be processed with other wastes, as it was mentioned in our previous paper [1], where tomato waste were blended and pelletized with sawdust.

Finally, in an increasing competitive context for the Tunisian agricultural sector, where the circular economy has become a frontline issue, the combustion of tomato waste can be an advantage in the tomato processing industries. Besides, a better environmental management of tomato by-products requires a better understanding of the economics of these wastes processing for bio-fuel production and for resolving issues related to the capabilities of combustion in practical applications. In this vein, the results of this study would serve as support in the manufacture of reactors in the first place, and in facilities of higher production in a second place.

## Figures and Tables

**Table 1:** Physical and chemical properties of tomato waste.

Parameter	Jeguirim et al. [6]	Kraiem et al. [1]	Jeguirim et al. [7]	Mangut et al. [8]	Yargıç et al. [9]
Moisture (%, wb)	10	10	8	4.22	7.18
Ash (%, db)	11	11	8	4.58	4.49
CF (%, db)	-	-	8	12.51	10.98
Volatiles (%, db)	-	-	76	78.68	77.35
ρ (kg·m^−3^)	52.2	52.2	50	-	-
HHV (MJ·kg^−1^)	19.5	19.5	19.5	22.4	20.47
Energy density (GJ·m^−3^)	10.2	10.2	9.75	-	-
N (%, db)	1.5	1.5	1.6	2.41	3.78
S (%, db)	0.29	0.3	0.35	0.038	-
K (%, db)	0.03	0.03	-	-	-
C (%, db)	-	54.2	59.4	49.52	49.69
H (%, db)	-	7	7.6	6.74	7.43
O (%, db)	-	20.2	23.4	-	39.1
Cl (g·kg^−1^)	5.75	-	-	-	-
Ca (g·kg^−1^)	1.45	8.1	-	-	-
Si (g·kg^−1^)	0.19	1.067	-	-	-
Na (g·kg^−1^)	0.35	1.932	-	-	-
P (g·kg^−1^)	0.93	5.1919	-	-	-
Mg (g·kg^−1^)	0.59	3.311	-	-	-
Al (g·kg^−1^)	0.12	0.641	-	-	-
Fe (g·kg^−1^)	0.10	0.556	-	-	-
Mn (g·kg^−1^)	0.09	0.05	-	-	-

**Table 2:** Fatty acid Composition of biomass, obtained by mechanical extraction (%W/W db).

Fatty Acid Composition	Tomato Seeds [13]	Olive Seeds [14]	Grape Seeds [15]
Palmitic acid C 16:0	14	11.5	6.6–11.6
Stearic acid C 18:0	5	2.5	3.5–5.4
Oleic acid C 18:1	21	75.5	14.0–20.9
Linoleic acid C 18:2	57	7.5	61.3–74.6
Linoleinic acid C 18:3	1	1	0.3–1.8
Myristic acid C 14:0	-	0	0–0.17
Anarchic acid C 20:0	-	0.5	0.1–1.7
Others	2	-	-

**Table 3:** Standard methods of physical and chemical characterization.

Parameter	Analytical Method
Sample Preparation	UNI EN 14780:2011
Moisture content	UNI EN 14774:2009
Ash	UNI EN 14775:2010
HHV, LHV	UNI EN 14918:2010
C, H, N, S, O	UNI EN 15104:2011

**Table 4:** Examples of kinetic models f(X) and their integral forms g(X).

Model	f(X)	g(X)
1st order	1 − X	−ln(1 − X)
Pseudo nth order	(1 − X)^n^	[1/(n−1)][(1 − X)^(1−n)^ − 1]
Contracted cylinder	2(1 − X)^1/2^	1 − (1 − X)^1/2^
Contracted Sphere	3(1 − X)^2/3^	1 − (1 − X)^1/3^
Energy law	νX^(ν−1)/ν^	X^1/ν^
Avrami-Erofe’eve	p(1 − X)[−ln(1 − X)]^(p−1)/p^	[−ln(1 − X)]^1/p^
Extended Prout-Tompkins	(1 − X)^n^X^m^	No analytical solution
1D diffusion	½X − 1	X²
2D diffusion	[−ln(1 − X)]^−1^	(1 − X)ln(1 − X) + X
3D diffusion (Jander)	[3/2(1 − X)^2/3^][1 − (1 − X)^1/3^]^−1^	[1 − (1 − X)^1/3^]^2^
3D diffusion (G–B)	3/2[(1 − X)^−1/3^− 1]	1 − 2X/3 − (1 − X)^2/3^

g(X) is the integral function of $\mathrm{Ln}\frac{\mathrm{dX}}{f\left(X\right)}.$

**Table 5:** Isoconversional Kinetic methods used in evaluating activation energy study.

Method	Expression	Plots	Ref
Friedman	$\mathrm{Ln}\frac{\mathrm{dX}}{\mathrm{dt}}=\mathrm{Ln}\left(\beta \frac{\mathrm{dX}}{\mathrm{dT}}\right)=\mathrm{Ln}\left[\mathrm{Af}\left(X\right)\right]-\frac{\mathrm{Ea}}{\mathrm{RT}}$	$\mathrm{Ln}\left(\beta \frac{\mathrm{dX}}{\mathrm{dT}}\right)$ vs. $\frac{1}{T}$	[21]
FWO	$\mathrm{Ln}\beta =\mathrm{Ln}\frac{\mathrm{AEa}}{g\left(X\right)R}-2.315-\left(\frac{1.0516\mathrm{Ea}}{\mathrm{RT}}\right)$	Lnβ vs. $\frac{1}{T}$	[22]
KAS	$\mathrm{Ln}\frac{\beta }{T^{2}}=\mathrm{Ln}\frac{\mathrm{AR}}{\mathrm{Eag}\left(X\right)}-\frac{\mathrm{Ea}}{\mathrm{RT}}$	$\mathrm{Ln}\frac{\beta }{T^{2}}$ vs. $\frac{1}{T}$	[23]

**Table 6:** Proximate analysis, ultimate analysis and energy content.

Water Content (%)	Volatiles (%)	Fixed Carbon (%)	Ash (%)	ρ (kg/m^3^)	LHV (MJ/kg)	Energy Density (MJ/m^3^)	C (%)	H (%)	O (%)	N (%)	S (%)
8	76	8	8	50	19.5	975	54.2	7	20.2	1.5	0.3

**Table 7:** Activation energies Ea (kJ/mol) and correlation coefficients R² calculated by KAS, FWO and Friedman models.

Model	KAS		FWO		Friedman	
X	Ea	R²	Ea	R²	Ea	R²
10	132.53	0.95	125.18	0.96	134.30	0.88
15	145.79	0.88	146.78	0.80	168.80	0.92
20	155.50	0.82	156.10	0.84	148.15	0.84
25	152.14	0.81	149.98	0.83	170.05	0.86
30	174.77	0.88	186.46	0.83	169.62	0.89
35	206.93	0.84	190.40	0.87	194.37	0.91
40	195.81	0.94	195.15	0.94	195.67	0.99
45	156.14	0.80	128.68	0.81	125.58	0.64
50	165.27	0.86	150.52	0.93	186.08	0.99
55	492.12	0.96	449.40	0.99	500.48	0.83
60	494.97	0.84	480.89	0.84	-	-
65	447.47	0.63	437.32	0.85	-	-
70	380.93	0.79	372.82	0.80	-	-
75	200.77	0.98	201.59	0.98	-	-
80	112.88	0.99	118.22	0.99	-	-
85	85.38	0.96	92.38	0.97	-	-
Average	231.21		223.87		199.31	
